# Mind the Gap: Gap Affordance Judgments of Children, Teens, and Adults in an Immersive Virtual Environment

**DOI:** 10.3389/frobt.2019.00096

**Published:** 2019-10-15

**Authors:** Sarah H. Creem-Regehr, Devin M. Gill, Grant D. Pointon, Bobby Bodenheimer, Jeanine K. Stefanucci

**Affiliations:** ^1^Department of Psychology, University of Utah, Salt Lake City, UT, United States; ^2^Department of Electrical Engineering and Computer Science, Vanderbilt University, Nashville, TN, United States

**Keywords:** affordances, perception, action, virtual environments, children

## Abstract

Affordances are possibilities for action that depend on both an observer's capabilities and the properties of the environment. Immersive Virtual Environments (IVEs) have been used to examine affordances in adults, demonstrating that judgments about action capabilities are made similarly to the real world. However, less is known about affordance judgments in middle-aged children and adolescents in IVEs. Differences in rate of growth, decision criteria, and perceived risk could influence affordance judgments for children. In Experiment 1, children, teens, and adults stood in an IVE at ground level or at a height of 15 m, and were asked to view gaps of different widths. Across all age groups, estimates of gap crossing were underestimated at the higher height compared to the ground, consistent with reports of fear and risk of falling. Children, compared to adults, underestimated their maximum crossable gap compared to their actual crossable gap. To test whether this difference was specific to IVEs or a more generalized age effect, children and adults were tested on gap estimates in the real world in Experiment 2. This real world study showed no difference between children and adults, suggesting a unique contribution of the IVE to children's affordance judgments. We discuss the implications for using IVEs to study children's affordances.

## Introduction

The utility of immersive virtual environments (IVEs) for many applications increases when viewers perceive the scale of the environment and their potential for actions within that environment similarly to the real world. Systematic study of human performance in IVEs over the last 15 years has led to a greater understanding of how adults perceive and act in IVEs. This work has historically included studies of distance and size perception (Thompson et al., 2004; Grechkin et al., 2010; Lin et al., 2011; Kelly et al., 2013), as well as more recent examinations of *affordances*, the perceived possibilities for actions in one's environment (Geuss et al., 2010; Bodenheimer and Fu, 2015; Creem-Regehr et al., 2015a; Jun et al., 2015). Affordances are critical for the study and enhancement of IVEs from an applied perspective because they can give an objective indication of how closely one's experience in IVEs match that of the real world (e.g., Geuss et al., 2010). Limited research has been conducted with children in head-mounted-display (HMD) IVEs because it has only been recently, with new commodity-level HMDs, that accessible technology has become available to test children. Although large screen-based displays have allowed for study of perception and action in immersive environments with children, it is important to extend this work to immersive HMDs where no visual information for the real body is present. It is possible that children would act and interact within HMDs in ways that are different than with large screen displays because of different levels of reliance on body based information. Given the critical role of the body in affordances and the rapid change in children's bodies with development, as well as their potential to assess risk differently, our aim was to determine (1) whether children or teens differ from adults in decisions about gap-crossing affordances within an IVE and (2) whether an environmental context, which has been shown to affect adults' perceived risk (judging gap crossing from a tall height) (Geuss et al., 2016), would differentially affect children.

## Affordances are Body-Scaled in the Real World

Gibson's theory of affordances is based on the notion that perception and action are intimately linked, and that perception is best understood in its relevance to a viewer's own action capabilities (Gibson, 1979). Affordances are defined by the match between the actor's body characteristics and the features of the environment relevant for action. Early work based on this theoretical perspective assessed perception by measuring actions performed toward objects and features of the environment or estimates of these actions. For example, Warren and Whang (1987) demonstrated a reliable relation between the size of an aperture that adult participants passed through to the size of participants' shoulder widths. Furthermore, when observers were asked to report the width of an aperture that was just passable, this estimate was also scaled systematically to their shoulder width, leaving a small margin of error. This finding, as well as numerous others using different environmental features and actions (e.g., stepping, sitting, reaching), indicate that adults' estimates of their action capabilities are reliably scaled to their own body dimensions (Warren, 1984; Mark, 1987; Mark et al., 1990), which include both body-parts (e.g., arm length) and intrinsic information about the body such as eye height (Wraga, 1999). It is sometimes unclear as to which body part or intrinsic dimension should be used to scale affordances, so scaling to multiple dimensions is desirable (Warren, 1984). Evidence for these mechanisms has been further supported by studies in IVEs (as described in the next section) that allow for manipulation of body characteristics in ways that were more difficult or impossible in the real world.

Much of the work on children's affordances has focused on infants or young children, showing that increased physical experience with action improves the accuracy of decisions about actions (Franchak et al., 2010), and also supports the claim that actions and decisions about actions are influenced by physical body capabilities. For example, 14-month-old infants' decisions about walking down slopes changed when they were wearing heavier vs. lighter weights (Adolph and Avolio, 2000). Other studies comparing infants through early childhood have shown that affordance judgments for fitting hands through apertures were less accurate for children 5 years old and younger, compared to 7-year-olds and adults (Ishak et al., 2014). The younger children were more likely to attempt to fit their hand through an aperture that was too small, compared to the 7-year-old children and adults. Children between 6 and 8 years old were shown to overestimate their abilities in reaching, stepping across, and sliding under, for tasks that were well beyond their ability, and adults also overestimated their abilities in these tasks when they were just beyond their ability (Plumert, 1995; Plumert and Schwebel, 1997). In a locomotion task, 7-year-old children have also been shown to select paths around an obstacle that deviate toward the side with more space, similarly to adults (Hackney et al., 2014). However, unlike adults, children were inconsistent in the biomechanical factors (e.g., stepping strategies) used to guide path selection. In contrast, for stair-stepping affordances, 7-year-old children's judgments of maximum stepping height relied on a similar ratio of leg-length and stair-height as young adults (Cesari et al., 2003). For middle-aged children, a study of passing through apertures showed that 8- to 10-year-old children require a greater width (larger margin of safety) for deciding when to turn their shoulders to pass through, suggesting that children's affordances may not scale to their body dimensions for passing through in a similar way as adults (Wilmut and Barnett, 2011). In other studies of passing through, younger children, aged 4–7 years, judged they could pass through doorways smaller than their actual capabilities, whereas older children, aged 8–11 years, and adults did not overestimate their abilities in this way (Franchak, 2019). In manipulations of feedback and altered body size with a backpack, Franchak (2019) also showed that recalibration from practice feedback improves with age. The take-home message from this body of work suggests that children's judgments of affordances are different than adults' judgments. Children do not judge their actual capabilities as accurately; their affordance judgments appear to undergo substantial developmental change. Together, the literature suggests there are still open questions about accuracy, biases, and learning in affordance judgments in children at different ages. Variations in the type of affordance, as well as the consequences of error are likely contributing factors (Ishak et al., 2014).

## Affordances are Body-Scaled in IVEs

Generally, studies have found that adults are as accurate at making affordance judgments in virtual environments as they are in the real world. For example, in an IVE that was modeled to match a real environment as closely as possible, Geuss et al. (2010) found that adults' abilities to judge passability through an aperture did not differ when comparing virtual and real judgments. A follow-up study comparing multiple technologies including HMD and large-screen displays found that virtual judgments were similar to real world judgments when stereo viewing and viewpoint tracking with a screen-based display were active (Geuss et al., 2015). In addition, the ability to judge affordances for reaching through apertures or grasping cubes did not differ from the real world when stereoscopic information for depth was present in a desktop virtual environment (Stefanucci et al., 2015). Similarly, Regia-Corte et al. (2013) found that adults could reliably judge whether a virtual slanted surface could support upright stance and that variations in the surface material (ice or wood) led to changes in affordance judgments in an IVE.

IVEs are a unique tool with which to test the hypothesis that affordance judgments are scaled by body dimensions because they provide a means to manipulate body size with self-avatars or simply a means to remove visual information about the body if no self-avatar is present. The presence or absence of a first-person avatar has been shown to change the threshold of adult viewers' decisions for stepping off of a virtual ledge (Lin et al., 2013) and stepping over or under a bar (Lin et al., 2012). When avatar body size is explicitly manipulated in IVEs, affordance judgments change as would be predicted by body scaling. For example, enlarging virtual hand size led to adults' judgments that larger virtual objects could be grasped (Linkenauger et al., 2013) and viewing one's feet as a larger or smaller virtual size modulated judgments of gap crossing; adults with larger virtual feet judged they could cross larger gap widths than those with smaller virtual feet (Jun et al., 2015). Although these studies with adults suggest that affordances are body-scaled in IVEs, the variability as a function of task, age, and feedback in the previously described real-world studies suggests the importance of directly testing children in IVEs.

## Affordance Judgments are Influenced by Risky Context in IVEs

In addition to adults' use of their bodies to scale affordance judgments in IVEs, a number of studies have shown that a particular environmental context—an IVE that includes a height—also affects affordance estimates. Manipulating environments to make them more risky leads adults to make more conservative estimates of their abilities. This claim was first supported by research done in real environments. For example, Graydon et al. (2012) found that anxiety induced by a breathing manipulation reduced adults' perceptions of what they could reach through, reach to, and grasp. Even more relevant to the current study, Jiang and Mark (1994) found that adults' estimates of whether they could cross a gap became more conservative as the gaps were raised off the ground. They speculated that the change in estimates was due to fear induced by the environmental context, but they did not directly measure perceived risk or fear in their experiment.

Virtual reality provides an excellent medium for reliably manipulating perceived risk and for testing its possible effects on affordance judgments in children and teens. Early work that tested how different modes of locomotion in VEs (i.e., button-controlled flying vs. actual walking) influenced feelings of presence found that adults who actually walked to a ledge and then looked down over a room, or *the pit* as the authors called it, felt the greatest sense of presence (Usoh et al., 1999). A follow-up to this study also demonstrated consistent increases in adult participants' heart-rates as a result of looking over the pit (Meehan et al., 2002), with inclusion of a small ledge over the pit increasing heart-rate even more (Meehan et al., 2005). More recently, research has shown that more realistic rendering of a pit environment (e.g., with ray tracing and shadows) leads to a greater sense of presence and an increased heart rate compared to less realistic rendering techniques (Slater et al., 2009; Phillips et al., 2012). Thus, presenting a height in an IVE produces a reliable increase in presence and physiological changes associated with an increase in perceived risk. Using both a manipulation of height in an IVE and a trait measure of fear of heights to assess individual differences, Geuss et al. (2016) found that both state and trait fear of heights influenced adults' affordance judgments in a gap-crossing task when the gaps were presented at a height of 15 meters as compared to ground level. We modeled our current IVE after Geuss et al. (2016) to further test for a change in perceived risk in children and teens.

Much of the research on children's affordances using virtual reality has focused on *dynamic* environments in risky contexts, such as the task of crossing a street on a bicycle among continuous traffic, in contrast to the real-world static (and relatively safe) environment tasks (e.g., passing through a aperture) described above. Plumert, Kearney, and colleagues used a large-screen stereo display and a real-time bicycle simulator to test decisions about crossing gaps in traffic and corresponding timing of entry into the intersection (Plumert et al., 2004, 2011). They showed that 10- and 12-year old children made similar estimates as adults for crossing a single lane of traffic in terms of gap widths, but left substantially less time for themselves to cross the gap compared to adults (Plumert et al., 2004). Children at these developmental stages did not choose larger gaps to compensate for their timing in crossing, suggesting that decisions about action and physical timing of actions are not completely consistent.

Studies using this paradigm have also shown that experience with crossing intersections changed both children's and adults' crossing decisions and behaviors (Plumert et al., 2011; Chihak et al., 2014). Ten-year-olds showed the greatest amount of improvement in their movement timing, compared to 12-year-olds or adults (Plumert et al., 2011). Furthermore, specific experience with crossing high-density traffic led both children and adults to choose smaller gaps to cross and to adjust the timing of their actions to match these riskier decisions. However, the children were not as successful in accurate timing, resulting in more virtual collisions. In a recent study, O'Neal et al. (2018) extended this work to crossing virtual roads on foot, examining whether coordination between gap choices and timing of actual action improved with age. Six- to 10-year-old children chose to cross narrower gaps, resulting in more collisions than 14-year-olds and adults. Interestingly, 12-year-olds indicated poorer timing of actions, but compensated by adjusting their gap crossing to choose wider gaps, suggesting that with development into adolescence, children begin to learn to match their decisions to their actual action capabilities.

The work described here suggests that, between the ages of 10 and 12, tuning of perception-action abilities in the context of dynamic affordances continues to develop. Children within this age group may make decisions for street crossing based on similar visual information about gap width as adults, but their movements do not correspond to these judgments, leading to riskier behavior. Although these results provide valuable insight into predictions about performance on other tasks in IVEs, it is an open question as to how children's affordances in static and risky contexts, as previously demonstrated in the real world to rely on body-based scaling, compared to adult performance. Furthermore, the street-crossing affordance work relies primarily on a screen-based VR technology linked to real actions. Our goal was to specifically examine children's performance within a class of new commodity-level HMDs, which, because of low-cost and comfort, are likely to be increasingly used in numerous applications with children.

## Overview of Experiments

As is clear from the review above, adults' affordance judgments in IVEs are influenced by both risky environmental context and changes to body characteristics. However, whether these effects are different in children remains unknown. Real-world studies of children's judgments of affordances have shown mixed results in comparison to adults—whereas some show comparable scaling to body dimensions, others show decisions about actions that have been both more liberal (e.g., reaching through an aperture that is too small) or more cautious (e.g., allowing more space for passing through an aperture before turning) than adults. Further, the IVE affordance work shows that younger children tend to act in ways that are riskier (e.g., when crossing a street, they accept smaller temporal gaps between cars), and also shows developmental differences in decisions about actions between younger and older children. Thus, our goal was to create a task that involved both a risky environment context shown to affect perception in adults (Geuss et al., 2016) and an affordance that has been shown in adults to be scaled by actual body capabilities in IVEs (Jun et al., 2015). We compared performance across three age groups: children aged 9–12 years, teenagers 13–17 years, and young adult college students (mean age 22 years). We included the teenage group to tease apart effects of body size and physical body growth (Newell and Wade, 2018) as well as the potential for risky judgments. While affordances do depend on physical body scale, the development of perceptual-motor abilities is influenced by the timescale of physical body growth, which is non-linear and inconsistent across different body parts (Newell and Wade, 2018). Teens and adults may be essentially the same in body scale as measured by height and leg length, but the growth spurts experienced more recently by teenagers may influence their action capabilities and judgments of these capabilities. In Experiment 1, we implemented a virtual world that included gaps of different widths to cross, both on the ground and on a platform at 15 m above the ground. Experiment 2 was run as a follow-up experiment in the real world given that the results of Experiment 1 showed underestimation in children's estimates (when scaled to their actual step) relative to the other age groups. With the second experiment, we aimed to determine whether the results were specific to the IVE or generalizable to a real-world gap-crossing task.

### Experiment 1

Participants of three age groups were tested on estimates of gap crossing: younger children, teenagers, and young adults with the IVE. We also assessed their perceived risk during the experiment and obtained measures of body dimensions and real stepping abilities outside of the IVE. Based on prior work with gap-crossing estimates and heights, we predicted that all participants would judge smaller crossable gaps at the 15 m height relative to the ground. We also hypothesized age-related effects, where children would show differences from adults in their scaling of estimates to body parameters or capabilities because of rapid changes in body size that are occurring or have occurred recently. Finally, we predicted that age group and location of the platform (ground or height) could interact, showing that heights have a greater influence on adults because the younger age participants may be more likely to accept risky behavior.

## Methods

### Participants

Thirty-seven participants were recruited from three distinct populations. Young children (ages 9–12 years old) were recruited by advertising within the University of Utah community. A total of 12 (6 female, 6 male) children participated in the study (*M*_age_ = 10.4, *SD* = 1). Each child received $10 for his or her participation. Thirteen adolescents (6 female, 7 male, *M*_*age*_ = 15.6, *SD* = 1.32) were recruited from a local high school and also received $10 for their participation. Our adult sample consisted of 12 undergraduates (6 female, 6 male, *M*_*age*_ = 22, *SD* = 3.5) recruited via the University of Utah Department of Psychology participant pool. They received class credit for their participation. Parental consent was received for those participants under the age of 18 and all participants gave informed consent/child assent before participating.

### Materials and Design

**Figure 1:**
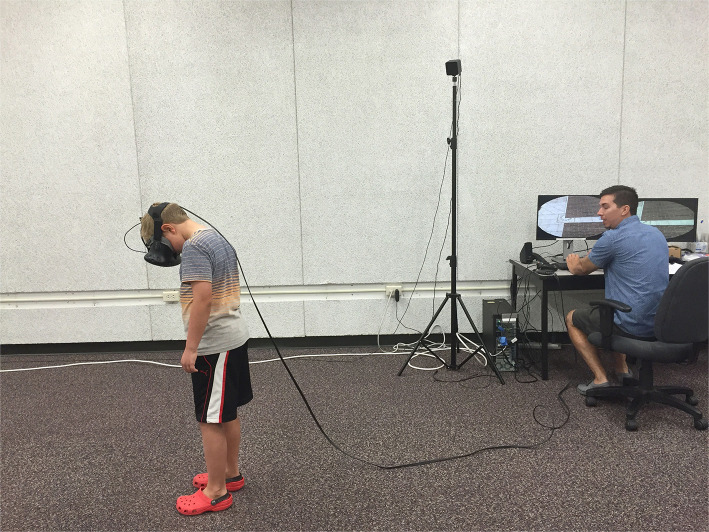
Child participant viewing the gap. Written informed consent was obtained from the individuals portrayed in this image.

**Figure 2:**
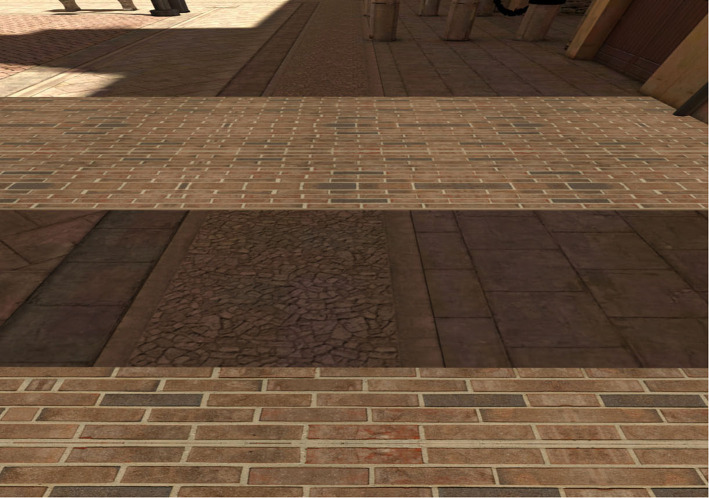
An example of a gap on the ground. The viewer stood with their toes at the edge of the near brick surface and judged stepping so that their heel would touch the far brick surface.

**Figure 3:**
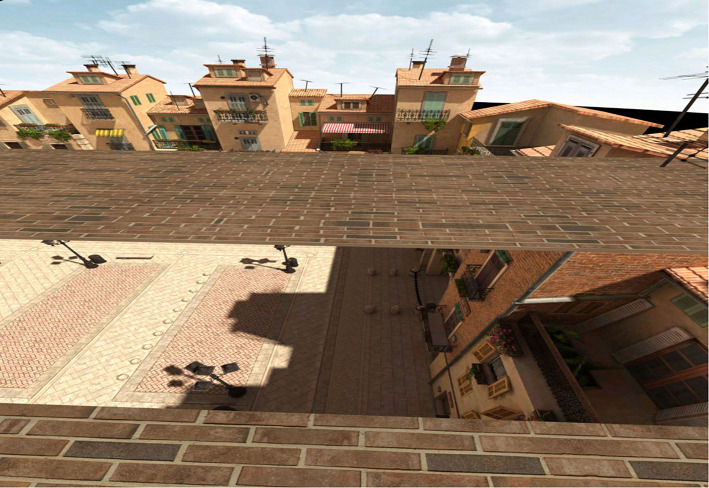
An example of a gap at 15 m height. The viewer stood with their toes at the edge of the near brick surface and judged stepping so that their heel would touch the far brick surface.

An HTC Vive head mounted display (HMD) was used to present the virtual environment to participants. The HTC Vive has a resolution of 1,080 × 1,200 pixels in each eye, weighs 1.22 lbs, and has 100° horizontal × 110° vertical field of view (Kelly et al., 2017). Participants' movement was tracked with two HTC Vive Lighthouse base stations. Figure 1 provides a picture of the Vive setup and a child participant. In the experiment, participants were placed in a virtual environment designed by WorldViz (an outdoor, rectangular Italian piazza). We modified this pre-programmed environment so that participants experienced the space on a virtual brick platform (45 m long, 2 m wide, 5 cm tall), similar to Geuss et al. (2016). The platform was placed on the ground or raised 15 m in the air (see Figures 2, 3). Another platform of equal size was presented across from the platform on which the participants stood. We manipulated the distance of the gap between the two brick surfaces in order to create the experimental trials. For adults and teenagers, we presented ten different gap widths that were between 0.55 and 1.45 m and varied by increments of 0.1 m (i.e., 0.55, 0.65, and 0.75 m, etc.). Based on informal preliminary tests with children, we designed the range of gaps that we presented to the children to begin and end at gap widths that were 30 cm shorter (0.25–1.15 m) than the adult/teen widths, to accommodate their average smaller stature and shorter leg length. All participants were presented with the 10 gap widths twice in both the ground and height conditions. The ground condition was always presented first, to serve as a baseline assessment before potential perceived risk was introduced.

We used a post-experiment questionnaire to examine differences in experiences related to VR and gaming. The four questions asked were: (1) How often do you play video games? (never, once a month, once a week, two or more times per week, everyday); (2) Have you ever used immersive Virtual Reality before? (yes/no); (3) How many times have you used Virtual Reality? (1 time, 2 times, 3 times, 4 times, 5 or more times); and (4) How comfortable did you feel in the head-mounted display? (I did not like it, I liked it but made me feel sick, I liked it but made me feel tired, I liked it and felt no problems).

Participants also completed a computerized version of the Corsi block-tapping task (Milner, 1972) after the gap estimates as part of another project that is not reported here.

### Procedure

Participants entered the lab and were given an overview of the experiment. All participants were told that they would judge whether or not they could step over virtual gaps of different sizes within the virtual environment. The experimenter explicitly stated to the participants that they should think about stepping as far as they could without feeling like they would fall or lose balance in order to make a yes or no judgment as to whether the gap was crossable. Then they were told that they would be asked to make this judgment from a stationary position and would not be allowed to actually step. For added understanding, the experimenter demonstrated the step to the participants, being careful to point out that one foot needed to be on the floor at all times (i.e., participants should not consider even small jumps in making their decisions).

Following the step practice, participants were given a detailed explanation of the experimental task. Each participant was told that they would enter a virtual environment using the HMD and that they would see gaps of different sizes for which they would have to make a yes/no decision as to whether or not they felt they could step over. Participants then donned the HMD. Once in the IVE, participants were allowed to move and look around to familiarize themselves with the space. After participants acclimated to the environment, they were guided to the brick platform on the outer edge of the piazza and asked to position themselves so that they felt like their toes were on the edge of the platform that faced the inner brick platform. From here, participants were told that they were allowed to move their torso and head but that they had to keep their feet fixed for the remainder of the experiment.

Before making judgments for each of the gap widths, participants answered three questions on a 0–100 Likert scale (see Geuss et al., 2016). These questions asked if participants were afraid of the height at which they were standing relative to the ground, whether they felt like they would fall if they attempted to step, and finally, how hurt they felt they would be if they did fall from their current standing position. These questions were asked twice throughout the experiment: once at the beginning of the ground-level block of trials and once at the beginning of the 15-m block of trials. After answering these questions, participants began the trials for judging the cross-ability of gap widths. On each trial participants verbally reported whether or not they felt that they could step across the gap with either a yes or no response. Once participants reported their judgments, the experimenter initiated the next trial. Between each trial, a red fixation cross on an all-black background appeared for 3 s. Participants were told to look up at the fixation cross while the next trial loaded. We did this in order to reduce the possibility that our participants experienced motion sickness from continuously looking down, and to prevent the participants from memorizing the visual image of the previous trial.

During the experiment we also recorded participants' eye height, physical height, hip height (measure from ground to self-identified hip bone) and step length (measured twice) from toes on a starting line to the heel of the extended foot (the toe to toe extent was also measured but not used in the analysis. We took these measurements either before or after the gap judgment task, depending on how many participants arrived in the lab at once. When participants arrived as groups the first participant of the group was run by one experimenter while the other experimenter collected the measurements listed above on the idle participants. Although these were recorded at different times, all participants practiced the step before the measurements were collected.

## Results

### Calculating Gap-Crossing Estimates

In order to assess the perceived affordance for each individual and his or her body size, we first calculated a *crossover* point, or the largest gap width at which participants said that they could no longer step over, but for which they had indicated that they could step over all smaller gaps. In order to reliably determine this gap width, we evaluated their responses to the gap widths in ascending order. The point at which their responses switched from “yes” to “no” reliably (i.e., two or more continuous no responses) was chosen as the crossover point. At this point, the average width between the smallest “no” response and the largest “yes” response was calculated. The crossover point was then scaled to actual maximum crossable gap (crossover/actual step length measured to heel). A ratio value of 1 means that participants' estimated maximum crossable gap width is equivalent to their actual maximum crossable gap as measured by their step in the real world. Ratios larger than 1 indicate that participants overestimated their ability, and ratios smaller than 1 indicate that participants underestimated their ability. Given that measures of actual step capability can be influenced by factors beyond objective body dimensions (e.g., strength, motivation, context) and these might also differ by age, we also created ratios of estimates to the measured eye height, to normalize gap estimates relative to body size. Jiang and Mark (1994) provide a model of how gap width is scaled in terms of user eye height and this approach has been used in scaling affordance estimates in past research (Mark, 1987; Geuss et al., 2016). These calculations resulted in 4 crossover point ratios (ground and height estimates each scaled to actual crossable gap and eye height) per participant.

### Body Dimensions

First, we compared body dimensions among age groups to see if differences did exist. Table 1 displays means and SDs of hip height, eye height, height, and actual crossable gap for each of the age groups. Univariate ANOVAs were run on each body dimension. For each of hip height, eye height, and physical height, children's dimensions were significantly lower than both teens and adults, who did not differ from one another, *ps* < 0.001. We also examined correlations between eye height and actual crossable gap (measured toe to heel) for each age group, adults, *r*_(10)_ = 0.57, *p* = 0.05, teens, *r*_(11)_ = 0.81, *p* = 0.001, children, *r*_(10)_ = 0.45, *p* = 0.15; as well as correlations between hip height and actual crossable gap, adults *r*_(10)_ = 0.57, *p* = 0.056, teens, *r*_(11)_ = 0.65, *p* = 0.02, children, *r*_(10)_ = 0.19, *p* = 0.55). Interestingly, children did not show significant correlations between their actual step and these body dimensions, whereas teens and adults (marginally) did.

**Table 1:** Means (SD) of body dimensions and actual crossable gap in cm for Experiment 1.

**Age group**	**Hip height**		**Eye height**		**Height**		**Actual crossable gap (toe-to-heel step)**	
Adults	102.58	(6.26)	161.29	(9.71)	172.29	(9.57)	88.44	(19.11)
Teens	104.46	(5.46)	162.19	(7.03)	173.15	(7.64)	96.96	(14.31)
Children	86.17	(6.46)	134.83	(6.95)	145.88	(7.47)	82.23	(15.86)

### Verifying the Manipulation of Risk

Our first hypothesis was that we would observe, across all age groups, more conservative judgments of crossing ability (underestimation of ability) at the height compared to the ground because of perceived contextual risk. We also hypothesized that children and teens might be more liberal in their judgments of what they could do (showing less of a difference between ground and height judgments) given that children exhibit less precise tuning of actions in risky contexts (O'Neal et al., 2018). In order to test these hypotheses, we first verified that our IVE was perceived as riskier when participants made judgments from a height of 15 m, and whether this perception of risk varied with age. Thus, responses to the three questions asked at the beginning of each block of trials (ground level and 15 m) were compared to assess whether or not we effectively manipulated perceived risk with the IVE. As a reminder, the three questions were as follows, answered on a 0–100 scale: “How afraid are you of the height that you are at?;” “How likely are you to fall if you attempt to step across the gap from where you are at?;” and “How hurt would you be if you fell from the height that you are at?” Table 2 displays the means and SDs of question responses for each height condition and age group.

**Table 2:** Means (SD) of risk questions in Experiment 1.

**Height**	**Age group**	**Fear**		**Likelihood of falling**		**Injury**	
Ground	Adults	0.58	(1.44)	7.25	(17.11)	4.00	(4.63)
	Teens	0.23	(0.60)	2.39	(3.55)	3.85	(3.96)
	Children	1.17	(1.85)	2.83	(3.16)	2.67	(3.26)
15 m Height	Adults	43.92	(35.72)	25.58	(32.44)	88.33	(26.14)
	Teens	51.92	(18.55)	18.85	(14.85)	85.77	(16.69)
	Children	44.83	(19.36)	12.75	(20.88)	66.33	(28.39)

We ran three separate ANOVAs to compare responses to the questions across age group and height condition in order to verify that risk increased at the height and to see whether it did so for all age groups. A 2 (height) x 3 (age group) repeated measures ANOVA was performed on each question to examine the influence of height and age on perceived risk in the IVE. Importantly for our manipulation check, there was a main effect of height for each question. Participants indicated higher fear at 15 m (*M* = 46.89, *SE* = 4.21) compared to the ground level (*M* = 0.66, *SE* = 0.23), *F*_(1, 34)_ = 119.11, *p* < 0.001, $\eta_{p}^{2}$ = 0.78. There was no significant main effect of age on indicated fear, *F*_(2, 34)_ = 0.32, *p* = 0.73, $\eta_{p}^{2}$ = 0.02, supporting the claim that fear judgments did not vary across age groups. For the likelihood of falling question, participants indicated they were more likely to fall at 15 m (*M* = 19.06, *SE* = 3.89) compared to the ground level (*M* = 4.16, *SE* = 1.66), *F*_(1, 34)_ = 20.54, *p* < 0.001, $\eta_{p}^{2}$ = 0.38. Age did not significantly influence perceived likelihood of falling, *F*_(2, 34)_ = 1.01, *p* = 0.38, $\eta_{p}^{2}$ = 0.06. Finally, participants indicated higher scores related to possible injury from falling at 15 m (*M* = 80.15, *SE* = 3.96) compared to the ground level (*M* = 3.50, *SE* = 0.66), *F*_(1, 34)_ = 379.60, *p* < 0.001, $\eta_{p}^{2}$ = 0.92. There was a marginal main effect of age on perceived injury, *F*_(2, 34)_ = 3.18, *p* = 0.054, $\eta_{p}^{2}$ = 0.16. *Post-hoc* pairwise comparisons exploring this effect revealed that children (*M* = 34.50, *SE* = 3.60) indicated significantly less perceived injury than both adults (*M* = 46.17, *SE* = 3.60, *M*_*Dif*_ = −11.67, *p* = 0.028) and teens (*M* = 44.81, *SE* = 3.45, *M*_*Dif*_ = −10.31, *p* = 0.046). Adults and teens did not differ (*p* = 0.79). The interaction between height and age was not significant for any of the ANOVAs, again suggesting that while height had an overall effect on all three questions relating to perceived risk, this effect was consistent across age groups.

### Scaling Affordances to Actual Crossable Gap

**Figure 4:**
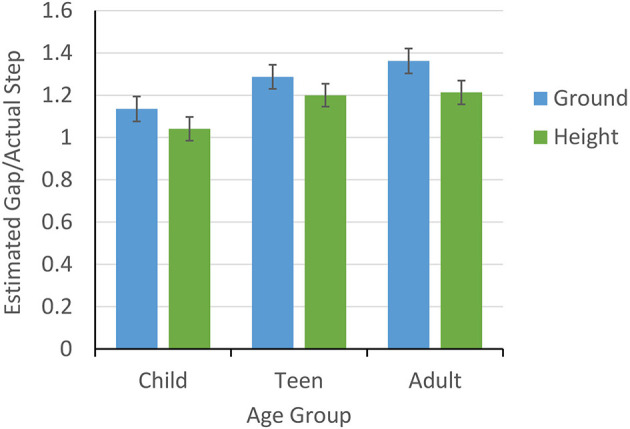
Mean (±1 SE) gap estimates scaled by actual step for child, teen, and adult age groups.

To assess the effect of height and age on crossover ratios scaled to participants' maximum crossable gap (step length measured toe to heel), we conducted a 2 (height) x 3 (age group) repeated measures ANOVA on the ratios with height (ground or 15 m) as a within-subjects variable and age group (children, teen, adult) as a between-subjects variable. Figure 4 shows a significant effect of height, showing significantly larger estimates on the ground (*M* = 1.26, *SE* = 0.03) compared to at 15 m (*M* = 1.15, *SE* = 0.03), *F*_(1, 34)_ = 17.16, *p* < 0.001, $\eta_{p}^{2}$ = 0.34. There was also a significant effect of age group, *F*_(2, 34)_ = 3.99, *p* = 0.03, $\eta_{p}^{2}$ = 0.19. Tukey HSD *post-hoc* comparisons showed lower estimates for children (*M* = 1.09, *SE* = 0.05) vs. adults (*M* = 1.29, *SE* = 0.05) (*M*_*Dif*_ = −0.20, *SE* = 0.07, *p* = 0.03 but not teens (*M* = 1.24, *SE* = 0.04) (*M*_*Dif*_ = −0.16, *SE* = 0.07, *p* = 0.1). Adults and teens were not different (*M*_*Dif*_ = 0.04, *SE* = 0.07, *p* = 0.82). There was no age x height interaction (*p* = 0.58). Overall, our results support the claim that although all participants overestimated their gap-crossing capabilities, younger children's affordance judgments were underestimated relative to adults', and all age groups estimated greater gap-crossing capabilities at the ground relative to the height. We did not find that age interacted with height, suggesting that increases in perceived risk affected the gap-crossing estimates of all age groups similarly.

### Scaling Affordances to Eye Height

**Figure 5:**
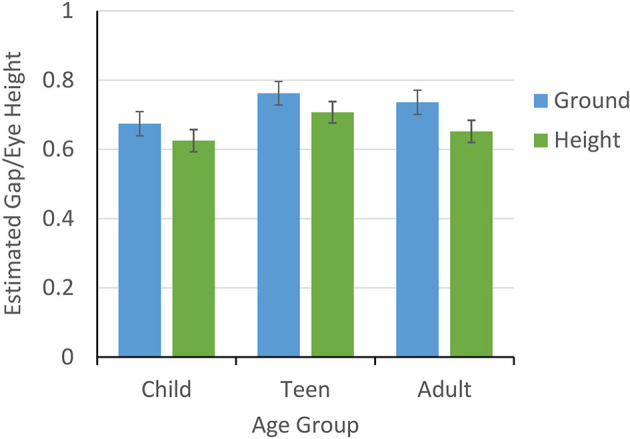
Mean (±1 SE) gap estimates scaled by eye height for child, teen, and adult age groups.

We repeated the 2 (height) x 3 (age group) repeated measures ANOVA using the crossover ratios scaled to participants' eye heights, as in Geuss et al. (2016). As in the previous analysis, there was a significant main effect of height, *F*_(1, 34)_ = 18.07, *p* < 0.001, $\eta_{p}^{2}$ = 0.347. Specifically, ratios were significantly larger at the ground level (*M* = 0.72, *SE* = 0.02) as compared to the 15-m height (*M* = 0.66, *SE* = 0.02), suggesting again that all participants were more conservative in their estimates of how far they could step when standing at a height (see Figure 5). Consistent with the direction of the ratios scaled to maximum actual crossable gap, children showed lower mean ratios scaled to eye height than the other groups, but the age effect did not reach significance, *F*_(2, 34)_ = 1.93, *p* = 0.16. There was no interaction between height and age.

### Individual Differences

We also asked participants four questions about their gaming and VR experience after completing the affordance judgments task. Only one adult reported playing video games every day, whereas 66% played only once a month or never. Roughly 82% of teens reported playing video games at least once a month or more, with more than 50% playing at least two or more times per week. Finally, 92% of children reported playing video games at least once a week or more, with more than 83% playing at least two or more times per week. Ten of our adult participants (83%) had never used immersive virtual reality before the experiment, whereas six teens (46%), and six children (50%) had previously experienced VR. The adult participants who had experienced VR reported using it at least three times prior to the experiment. Most teens and children had only experienced VR one time prior to the experiment. We tested the correlation between the frequency of video game play and gap-crossing estimates scaled to actual step at both the ground and 15 m height. The results showed that as frequency of video game play increased, the overestimation of gap crossing capabilities decreased at 15 m, *r*_(35)_ = −0.46, *p* = 0.004. A similar negative relationship was found for judgments at the ground, but it did not reach significance, r_(35)_ = −0.28, *p* = 0.094. These findings suggest that video game experience may affect accuracy of affordance judgments, although this relationship may be influenced by the overall higher frequencies of play seen in the younger children.

A high percentage (83%) of all participants indicated that they enjoyed the HMD and experienced no problems while wearing it. Only six participants reported feeling either tiredness (11%, one adult, two teens, and one child) or sickness (6%, one adult and one teen) as a result of wearing the Vive head-mounted-display. These results suggest that, across ages, participants did not experience differences in the IVE or wearing the head-mounted display and most enjoyed it.

## Discussion

When assessing judgments in the IVE relative to actual step in the real world, all groups showed some level of overall overestimation of abilities, consistent with previous real world studies of stepping and reaching (e.g., Plumert, 1995). However, the younger children showed less overestimation compared to teenagers and adults. When scaling the gap crossing estimates to eye-height, a more objective measure of body size, we found a similar trend of relative lower estimates of gap-crossing ability in children compared to the older groups, but the difference did not reach statistical significance. One explanation for the different conclusions across the two different ratios could be that children's actual largest steps did not strongly relate to their measured body dimensions. In fact, teens' and adults' actual measured step correlated more strongly to their eye-height and hip height compared to children. This lack of relationship could have been because of differences in the children vs. the other age groups in their interpretation or motivation of a “largest possible step.” We discuss the importance of serious consideration of scaling of affordance estimates, especially in the context of developmental differences, in the General Discussion.

All age groups also showed similar relative underestimation when judging affordances at a 15 m height compared to judging while on the ground. This finding replicated Geuss et al. (2016) and is consistent with our recorded subjective responses of increased fear and chance of falling, and a large body of work showing that IVEs can evoke behavioral and physiological responses associated with perceived height off the ground (Meehan et al., 2002; Slater et al., 2009; Phillips et al., 2012). The similar effect of height across age group did not support our prediction that children or teens might show riskier behavior as has been seen in previous work in dynamic road-crossing scenarios (Plumert et al., 2004, 2011).

Finally, given that the study was run completely with the IVE, the results of this experiment leave open the question of whether the difference seen in children vs. adult crossing judgments is a result of perceived affordances in IVEs, or more generally a developmental difference that would also be apparent in the real world. To address this question, we ran Experiment 2 completely in the real world, closely matching the procedures used in the first experiment (but judgments were made only on the ground).

### Experiment 2

In order to further understand the age effect on the crossover ratios when calculated relative to actual crossable gaps we ran a real-world experiment on new groups of younger children (mean age 10.5 years) and adults (mean age 19.5 years). Our goal was to determine whether we would replicate the underestimation of gap crossing in children relative to adults in the real world, or whether the finding may be specific to factors associated with perception and action within an IVE. The second experiment also allowed us to pursue the discrepancy in age-related results found in Experiment 1 when estimates were scaled to eye height, as compared to actual step.

## Methods

### Participants

Participants were children (ages 9–12 years old) recruited by advertising within the University of Utah community, who visited the laboratory to participate in another study, but who did not participate in Experiment 1. A total of 12 (4 female, 8 male) children participated in the study (M_*age* = 10.58, *SD* = 1.08). Children received $10 for their participation. Our adult sample consisted of 12 undergraduates (6 female, 6 male, M_*age* = 19.50, *SD* = 1.62) recruited via the University of Utah Department of Psychology participant pool. They received class credit for their participation. Parental consent was received for those participants under the age of 18 and all participants gave informed consent/child assent before participating.

### Materials and Design

We created a real world gap by placing gray fabric on the ground that could be adjusted in 0.1 m increments using a hidden tape measure on the side of the fabric. A blue metal measuring stick was placed on the ground to indicate the far edge of the gap that the participant should think about stepping over, and the participant stood at a line of tape on the floor at the close edge of the gap. Gap widths were presented in a random order for each participant exactly as in Experiment 1 (range 0.55–1.45 m for adults and 0.25–1.15 for children).

### Procedure

Participants signed consent (and parents/guardians signed permission) at the beginning of the experimental session. The participant's age was recorded. Participants were told that they would be judging whether or not they could step over a gap on the ground in the real world, with their toes at the near edge and viewing the far edge in depth. Care was taken to use the same instructions and demonstration as in Experiment 1. The experimenter explained that the participant should imagine taking the biggest step possible without feeling as though they would fall or lose balance. The participant wore a blindfold while the experimenter set up the first trial. After the gap was set, the participants viewed the gap and answered “yes” if they decided they could step over the metal stick so that their heel would clear the far edge, or “no” if they could not. Participants then lowered the blindfold until the experimenter signaled that they were ready for the next trial. At the end of the experiment, the experimenter took the participant to a new location in the lab and measured the participant's actual largest step. The participant stood with toes at the edge of a tape line and took the largest step possible, following the instruction that they would keep their back foot on the floor and step without jumping or feeling as if they would lose their balance. This step was measured (from both the toe starting position at the initial line to both the heel and toe of the extended foot) and then was repeated a second time. After the completion of the steps the experimenter measured in cm the participant's hip height (from hip bone to floor), height (floor to top of head) and eye height (floor to center of eye).

## Results

### Body Dimensions

Table 3 displays means and SDs of hip height, eye height, physical height, and actual crossable gap for children and adults. Independent *t*-tests were run on each body dimension to compare children and adults. For eye height and physical height, children's dimensions were significantly lower than adults, *ps* < 0.001. The hip height difference was marginally significant (*p* = 0.08). As in Experiment 1, we tested the correlation between eye height and actual step for adults, *r*_(10)_ = 0.78, *p* = 0.003, and children, *r*_(10)_ = 0.07, *p* = 0.82; and we tested the correlation between hip height and actual step for adults, *r*_(10)_ = 0.68, *p* = 0.015, and children, *r*_(10)_ = −0.09, *p* = 0.79. Again, whereas adults showed significant relationships between these body dimensions and their step capability, children did not.

**Table 3:** Means (SD) of body dimensions and actual crossable gap in cm for Experiment 2.

**Age group**	**Hip height**		**Eye height**		**Height**		**Actual crossable gap (toe-to-heel step)**	
Adults	95.67	(5.82)	163.75	(11.05)	175.42	(10.39)	104.33	(24.93)
Children	90.54	(7.89)	135.88	(13.02)	146.96	(12.35)	86.44	(15.25)

### Crossover Ratios

**Figure 6:**
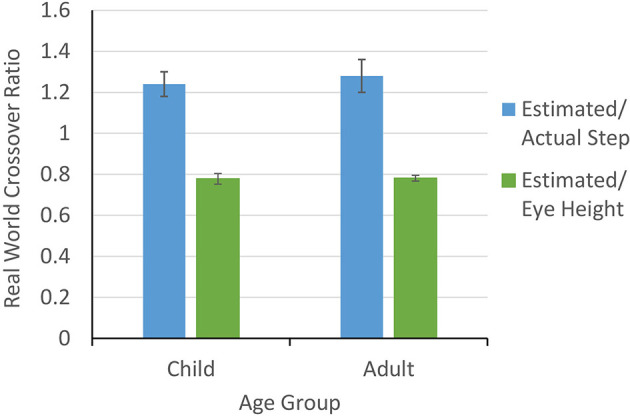
Ratios for stepping over a gap when scaled to actual step and eye height for both children and adults in Experiment 2.

We ran independent samples *t*-tests to compare crossover ratios across child and adult age groups, scaled to actual step (measured toe to heel) and eye-height. In contrast to Experiment 1, in the real world, we found no difference between children (*M* = 1.24) and adults (*M* = 1.28) in the crossover ratios scaled to actual step, *t*_(22)_ = −0.35, *p* = 0.73 (M_*diff* = −0.035, SE_*diff* = 0.10). There was also no difference between children (*M* = 0.78) and adults (*M* = 0.78) in the crossover ratios scaled to eye-height, *t*_(22)_ = −0.08, *p* = 0.94 (M_*diff* = −0.002, SE_*diff* = 0.03, see Figure 6).

## Discussion

Experiment 2 was run to test whether children's relatively lower gap-crossing estimates relative to actual step could be attributed to factors associated with the IVE or whether the result generalized to a real world environment. Children and adults performed real-world estimates of gap crossing using the same method as that run in the IVE (although in the lab environment rather than in the piazza IVE). Their estimates were scaled as crossover ratios to actual maximum crossable gap and to eye height. Both ratios support the conclusion that there was no difference between children and adult gap-crossing judgments in the real world. These results suggest children and older participants in Experiment 1 may have differed in their estimates due to factors associated with judging affordances within IVEs rather than an overall tendency for children to underestimate their abilities relative to adults. However, another notable difference between Experiment 1 and Experiment 2 is the greater average size of the adults' maximum actual step compared to that of Experiment 1. Larger measured actual steps would contribute to a lower crossover ratio (i.e., a larger denominator), possibly making the adult and children ratios appear more similar in Experiment 2. Likewise, if the adult actual steps in Experiment 1 had been larger, as seen in Experiment 2, there may not have been a significant underestimation in children relative to adults. Measured step size could be influenced by a number of factors including differences in experimenter instructions and participants' motivation, although the difference could also have been due to actual differences in the capabilities of the participants. Measured step size could have also differed based on the clothes that participants wore to the experiment. For example, females wearing skirts were not able to step as far. Regardless, the discrepancy in average step (when height and eye height were similar across the two samples) introduces an important factor to consider in the analysis of affordance judgments. Different conclusions may be reached depending on the way affordance estimates are scaled. These differences are further discussed in the next section.

### General Discussion

Our goal was to determine whether children and teens differ from adults in their judgments of gap-crossing affordances, and whether these judgments would be differentially affected by perceiving gaps at heights. Our first hypothesis was that children and teens, who are experiencing (or more recently have experienced) rapid growth and changes to their bodies, would differ in how they scale their estimates to their body capabilities. We found partial support for this hypothesis in that children's crossover ratios in the IVE, when scaled to their actual, maximum step, were lower than those of teens and adults, who did not differ from each other. The crossover ratios when scaled to eye height, showed a mean difference suggesting an effect in the same direction, but the difference did not reach statistical significance. Experiment 2 helped to elucidate this result as one specific to the IVE, as children and adults showed no difference in their estimates in the real world. However, it is possible that this lack of difference may have been due to the overall larger actual steps measured in Experiment 2. Together these findings suggest that crossing estimates in IVEs may be different for younger children compared to teenagers and adults, at least when measured relative to actual stepping ability.

Our second hypothesis involved effects of perceived affordances for crossing in a riskier context of a platform 15 m off the ground. Here, we found the predicted effect of more cautious estimates when made above the ground vs. on the ground, but we did not see a difference in this effect as a function of age group. All ages showed a similar underestimation of ability. These findings support the notion that IVEs can create contexts that evoke fear or stress similarly across different age groups, consistent with a history of work in IVEs such as pit rooms and walking on planks (Meehan et al., 2005) and more recent work with distance and gap estimates at virtual heights (Geuss et al., 2016). We discuss below several possible accounts for the body-scaling and height findings in the context of prior and future work.

### Body-Scaled Affordances for Gap Crossing in the IVE

We found that 9–12 year old children judged lower gap crossing abilities in an IVE when assessed relative to their actual demonstrated abilities, in comparison to teenagers and young adults. However, this age effect was not seen for the same affordance judgments in the real world and was not as large an effect when the estimates were evaluated relative to eye height. What underlies the difference seen in the IVE vs. real world for the younger children, specifically when scaled to their actual actions? One possibility is that children differ from adults in their abilities to relate visual information in the virtual environment to their actual actions but are closer to adults in the ability to implicitly interpret the visual information with respect to their eye height. In fact, the demonstration that children were relatively similar to the other age groups when assessing estimates in this way is promising for establishing that children can use eye height to inform perception of space within an IVE. Further, in scaling to actual step in Experiment 1, it is important to note that the step was performed in the real world, but judgments were made in the IVE. Thus, it could be that children were less able to translate their real world stepping performance to the IVE than teens or adults. Another reason why the younger children's judgments when scaled to actual step may have differed from the other groups is that they did not show consistent relationships between their body dimensions (eye height and hip height) and their step performance. Thus, while teenagers and adults showed correlations between their actual step abilities and measures of body size (i.e., those who were taller and had longer legs stepped farther), our samples of young children across two experiments did not show these correlations. Overall, we believe that assessing affordance estimates relative to two different types of body characteristics (i.e., both a physical capability and a body dimension) is a useful way to tease apart potential age differences in perception and action in IVEs, given that it is currently a relatively unexplored area of research. Given the large variation in actual performed steps across the two experiments, our research suggests the value of including a more “objective” measure of body size such as eye height, although scaling to this dimension does not allow for claims about relative accuracy to one's actual actions. It is notable that different conclusions can be reached based on whether the crossing estimates were scaled to actual step or eye height. With actual step, we suggest that children underestimate their capabilities in the IVE relative to adults; with eye height, there is not support for this difference.

Although we hypothesized that developmental differences in affordance estimates might be due to rapid body growth or inherent differences in body dimensions across age groups, it could also be the case that children perceive distances across gaps differently than teens and adults. We did not directly test for the perception of the size of gaps, as in Jun et al. (2015), but future studies should test for this possible difference in perception. The question of distance underestimation within HMDs has been examined extensively with adults, using the traditionally heavier and limited-field-of-view HMDs, as well as more recent comparisons to commodity-level HMDs. Recent consensus with commodity-level HMDs points to less underestimation of distance compared to what was traditionally found in early studies (Young et al., 2014; Creem-Regehr et al., 2015b; Kelly et al., 2017; Buck et al., 2018). Given that recent work still finds some distance compression in IVEs in the HTC Vive with adults (Kelly et al., 2017), perhaps children perceived even less compression than teens or adults, which would provide an alternative explanation for their more conservative affordance judgments (i.e., if children perceive the width of the gap to be farther, then they would choose a smaller interval as crossable). Just as this study provides much needed preliminary data about how children and teens may perceive affordances in IVEs, further experiments should be conducted to test children's perception of absolute distance and size in IVEs more broadly.

### Perception of Risk for Gap Crossing in the IVE

Given that prior work has shown children to be less conservative in a risky context such as street crossing (Plumert et al., 2004, 2011), the fact that the children in our study reported more conservative gap crossing estimates at the 15 m height, similarly to teens and adults, is interesting. Although these results seem to oppose one another, they could be explained by the amount of experience children have with each affordance. In the case of street crossing, one assumption is that children are typically guided or supervised by parents. Children may not have had enough independent street crossing experience to couple the optical information that specifies a 'safe' crossing with their own action capabilities. Indeed, if children are given practice crossing virtual streets, their ability to perceive when a street crossing affords safe passage as well as their action timing improves (Plumert et al., 2011; Chihak et al., 2014). Unlike street crossing, children have more experience stepping over gaps whether at the playground or when encountering obstacles in the environment. Greater experience would suggest that childrens' perceptual systems have learned the consequences of misperceiving gap crossings (i.e., falling), which could lead to the more conservative estimates we found at the 15 m height.

### Limitations and Future Directions

There are several methodological limitations to this work that should be taken into account when considering the conclusions and future directions. It is possible that the lower range of gap widths presented to the children compared to that used with the teenagers and adults contributed to the difference in gap estimates. Previous work has shown that children tend to overestimate their capabilities more when the intervals are slightly beyond their abilities (Plumert, 1995). Given that the post-experiment measurement of actual steps of the children were larger than expected, the lower range of widths led them to experience a greater proportion of smaller gap widths that fell well below their abilities, possibly explaining the reduced overestimation relative to the other age groups. Future work could take the approach of measuring actual step initially and adjusting the range of gap widths to the individual's own capabilities. However, this account of the difference between younger children and the other groups in Experiment 1 is not supported by the results of Experiment 2, where the same lower range of gap widths was used with children but there was no difference in their estimates relative to adults. Another limitation of this work is the cross-sectional design, which grouped children into younger and older categories of 4–5 years. As seen in the descriptive body data in Tables 1, 3, there is a good amount of variability in children's body dimensions and capabilities within their age group. Future work could take different approaches to address this variability, including grouping into smaller age increments and increasing sample size. Although more difficult, a longitudinal approach could also be taken, where the same children are tested over time (e.g., 6 month or 1-year intervals) to more precisely relate their developmental growth to their affordance judgments.

It is important to recognize that most of the previous affordance judgment studies with children have also assessed actual performance along with decisions about action. For example, judgments of when to cross a street in traffic in an IVE are made along with measuring the actual timing of movement (O'Neal et al., 2018). Likewise, judgments of passing through apertures in the real world are made in the context of actually walking through (Franchak, 2019). In our current work, we set up a context to induce risky action, and measured the decision to take action, without asking participants to perform the action itself. Measuring changing dynamics in the actions of gap crossing could provide additional insight into differences among age groups. It could also help to answer questions related to feedback and calibration, which are likely to vary across age in the IVE as they have been shown to vary in the real world (Franchak, 2019). Thus, future work on affordances in children both comparing virtual environments to the real world and implementing physical actions in the IVE is needed.

Finally, the paradigm used in our current study easily lends itself to the addition of self-avatars. Given the prior work on effects of virtual bodies and body-parts on embodiment (e.g., Kilteni et al., 2012; Steptoe et al., 2013; Kokkinara et al., 2015) and affordances within IVEs in adults (Lin et al., 2012, 2013; Linkenauger et al., 2013), it is a natural question to ask how children use and perceive self-avatars. Just as affordance estimates without avatars have been shown to vary in children here and in real-world studies, children are likely to use virtual self-representations of their bodies differently than adults. The effects of self-avatars on children could be predicted to result in behaviors in several possible directions. With the rationale that children's bodies are in continuous development, it could be argued that children would show even stronger effects than adults when given a body-size manipulation, because they have less stable current representations of their bodies. It could also be that because children are used to their bodies changing, they would not notice a visual change in their virtual body as much, leading to smaller effects.

## Conclusions

Our study demonstrates the feasibility of using a relatively new commodity-level HMD to test perception and action in children and teenagers. We found some support for the claim that children in middle childhood differ from adults in their affordance estimates, showing relative lower estimates for gap crossing in IVEs, relative to how they actually act in the real world. However, this difference only resulted when scaling estimates to actual step, and not to a measure of eye height, which was used as a more objective measure of physical body size. Additional studies are needed to test whether the relative underestimation of capabilities in children in IVEs is replicated with other affordances and other experimental designs and whether results may differ depending on the body characteristic or capability that is used as a metric. Our second novel finding is that judgments made from heights in an IVE affected children, teens, and adults similarly. All underestimated their abilities relative to their judgments on the ground, consistent with perceiving higher risk in their potential actions from the height. Future work should pursue the use of commodity-level HMDs to further investigate how children perceive other affordances in IVEs, particularly those that vary in level of risk, to generalize these findings. Furthermore, paradigms that include execution of actions along with estimates of capabilities, and presence of self-avatars will help in furthering our understanding of children's affordances. As the new HMDs become increasingly used for training and education, it will become even more critical to understand how users of all ages perceive and act within virtual spaces.

## Data Availability Statement

The datasets generated for this study are available on request to the corresponding author.

## Ethics Statement

The studies involving human participants were reviewed and approved by University of Utah Institutional Review Board. Written informed consent to participate in this study was provided by the participants or the participants' legal guardian/next of kin.

## Author Contributions

SC-R and JS conceptualized the project, analyzed data, and wrote and edited the manuscript. DG and GP collected data, analyzed data, and wrote and edited the manuscript. BB conceptualized the project and wrote and edited the manuscript.

### Conflict of Interest

The authors declare that the research was conducted in the absence of any commercial or financial relationships that could be construed as a potential conflict of interest.
